# Right Ventricular Systolic Dysfunction Predicts Recovery of Left Ventricular Systolic Function and Reduced Quality of Life in Patients With Arrhythmia‐Induced Cardiomyopathy

**DOI:** 10.1002/clc.70070

**Published:** 2025-02-22

**Authors:** Thomas Körtl, Franziska Mühleck, Paul Baum, Markus Resch, Christine Meindl, Ekrem Üçer, Lars S. Maier, Rolf Wachter, Samuel Sossalla, Christian Schach

**Affiliations:** ^1^ Medizinische Klinik I Justus‐Liebig‐Universität Gießen Gießen Germany; ^2^ Klinik und Poliklinik für Kardiologie Universitätsklinikum Leipzig Leipzig Germany; ^3^ Abteilung für Kardiologie Caritas Krankenhaus St. Josef Regensburg Germany; ^4^ Universitäres Herzzentrum Regensburg, Klinik und Poliklinik für Kardiologie Universitätsklinikum Regensburg Regensburg Germany; ^5^ Abteilung für Kardiologie Kerckhoff‐Klinik GmbH Bad Nauheim Germany

**Keywords:** arrhythmia‐induced cardiomyopathy, atrial fibrillation, quality of life, right ventricular function

## Abstract

**Introduction:**

Arrhythmia‐induced cardiomyopathy (AIC) is an underrecognized condition resulting in left ventricular systolic dysfunction (LVSD) that is primarily caused by atrial fibrillation (AFib). The relationship between AIC, right ventricular (RV) function, and quality of life (QoL) has not been well studied.

**Methods:**

We performed a post‐hoc analysis of our AIC trial in which we prospectively screened for patients with tachyarrhythmia and newly diagnosed, otherwise unexplained LVSD. Following rhythm restoration, patients were followed up at 2, 4, and 6 months. Only patients with persistent sinus rhythm were analyzed. RV function was assessed via echocardiography (tricuspid annular plane systolic excursion [TASPE] and fractional area change [FAC]) and QoL by the Minnesota Living with Heart Failure Questionnaire.

**Results:**

Of a total of 50 patients recovering from LVSD, 41 were diagnosed with AIC and 9 with non‐AIC. Initially, RV function was reduced in the AIC group and recovered after rhythm restoration, whereas no relevant changes were noted in the non‐AIC group. QoL was reduced in both groups and also improved after rhythm restoration. Regression analysis identified low TAPSE as a predictive parameter for AIC diagnosis and worse QoL in AIC patients.

**Conclusion:**

We demonstrated that RV function and QoL are impaired in patients with AIC. Six months after rhythm restoration, TAPSE may serve as an early indicator of AIC while also correlating with QoL. This underscores the importance of detailed echocardiographic evaluation with a focus on RV function in patients with concomitant tachyarrhythmia and LVSD.

## Graphical abstract

Initial tricuspid annular plane systolic excursion (TAPSE), quality of life (QoL) as measured by the Minnesota Living with Heart Failure Questionnaire and left ventricular ejection fraction (LVEF) during atrial fibrillation (AFib) or atrial flutter (AFlut) were reduced in patients with arrhythmia‐induced cardiomyopathy (AIC) compared with values at the end of 6 months of follow‐up in sinus rhythm. In this paper we show that low TAPSE (optimal cut‐off 18.5 mm) has good predictive power for the diagnosis of AIC and that a low quality of life is associated with low TAPSE. Values in red indicate the relative percent the baseline values were reduced compared with the post‐recovery measurement at the end of follow‐up.

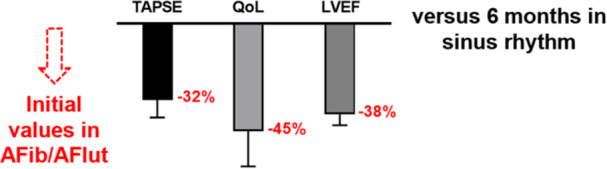

## 1 Introduction

Rhythm disorders with tachycardia and/or irregular heartbeat can cause arrhythmia‐induced cardiomyopathy (AIC), a subform of nonischemic dilated cardiomyopathy (DCM) [1]. Atrial fibrillation (AFib) is the most common arrhythmia that can trigger AIC and is present in up to 50% of patients with heart failure [2]. Until recently, little has been known about the incidence and time course of this potentially curable disease. Recently, we conducted a prospective study of patients with atrial tachyarrhythmia and otherwise unexplained left ventricular systolic dysfunction (LVSD) and showed for the first time that the incidence of AIC is surprisingly high in such a pre‐selected cohort and that left ventricular ejection fraction (LVEF) usually recovers within the first months after rhythm restoration [3].

AFib can be an underlying cause of LVSD as well as right ventricular (RV) dysfunction, which can be reversed after rhythm restoration [4]. In DCM, approximately one‐third of patients suffer from RV dysfunction, which is associated with a poor prognosis [5]. In addition, it is known that patients with DCM have a significantly reduced quality of life (QoL) [6]. In conditions other than AIC, such as systemic lupus erythematosus‐associated pulmonary arterial hypertension, QoL correlates with RV function as well as hemodynamic parameters and exercise capacity [7]. While there is now a better comprehension of LV function in AIC, the impact of AIC on RV function and QoL is poorly understood. Therefore, the aim of our study was to evaluate RV function and QoL in patients presenting with tachyarrhythmia and heart failure and to assess a potential association between RV function and QoL.

## 2 Methods

### 2.1 Study Design

In our original AIC study, patients were prospectively screened for LVSD and tachyarrhythmia (AFib or atrial flutter with a heart rate > 100/min) [3]. After initial assessment of left and right heart function, rhythm control was achieved and other causes of LVSD other than the arrhythmia per se were excluded as described previously in the main trial [3]. In brief, patients underwent coronary angiography, cardiac magnetic resonance imaging, blood sampling (cardiac stress markers), and QoL assessment by the Minnesota Living with Heart Failure Questionnaire (MLHFQ). Rhythm control was established by electrical cardioversion and/or electrophysiological ablation. Follow‐up evaluations at 2, 4, and 6 months after rhythm control that included electro‐ and echocardiography were carried out and QoL was measured. The main inclusion criteria were persistent AFib or atrial flutter, newly diagnosed LVEF < 50%, and initial heart rate > 100 beats/min. The main exclusion criteria were permanent AFib, valvular heart disease, suspicion of accumulative disease, suspicion of myocarditis, recurrent arrhythmia during follow‐up, relevant coronary stenosis in the current coronary angiography, and percutaneous coronary intervention or revascularization in the last 3 months. Patients with either an absolute increase in LVEF of ≥ 15% or by ≥ 10% with an absolute LVEF ≥ 50% were categorized as AIC; all others were classified as non‐AIC, thus representing a group with persistent cardiomyopathy. A detailed study protocol has been provided previously [8]. In this post‐hoc analysis, all patients who were included in our main trial were analyzed for RV function and QoL.

### 2.2 Echocardiography

The acquisition and analysis of the echocardiographic images were performed at the local study centers by experienced cardiologists using equipment from General Electric (GE), Solingen, Germany, or Philips Healthcare, Hamburg, Germany. RV function and dimension was assessed with standard 2D images in the apical four‐chamber view or subcostal view for inferior vena cava (IVC) according to current guidelines [9]. Only image loops with the highest quality were used to calculate LVEF by Simpson's biplane method. In atrial fibrillation, the mean LVEF of five consecutive cardiac cycles was used. Tricuspid annular plane systolic excursion (TAPSE) in the M‐mode was determined by measuring the longitudinal excursion of the lateral RV tricuspid annular plane during systole. Fractional area change (FAC) was calculated by using the equation (end‐diastolic area‐end‐systolic area)/end‐diastolic area × 100. Systolic pulmonary artery pressure (sPAP) was estimated by determining the peak velocity of the tricuspid regurgitation in continuous wave Doppler. The size of the IVC was assessed 2 cm distal to the hepatic vein takeoff. Basal right ventricular end‐diastolic diameter (RVEDD) and right atrial (RA) area at end‐systole were determined to evaluate RV dimensions.

Speckle‐tracking echocardiography was used to obtain RV longitudinal strain measurements in a semi‐automated fashion The RV was divided into 6 segments (basal, mid, apical, and for each free wall and septal segments). After generation of the strain curves, RV free wall longitudinal strain (RV‐FWLS) was calculated as the mean of the 3 RV free wall segments, and RV four‐chamber longitudinal strain (RV‐4CLS) was calculated as the mean of all 6 segments (Figure S1). Echocardiographic images were analyzed by one experienced investigator employing the IntelliSpace Cardiovascular software (Philips Medical Systems, Hamburg, Germany).

According to a consensus paper from the American Society of Echocardiography and the European Association of Cardiovascular Imaging, RV function was classified as being impaired when TAPSE was < 17 mm and FAC < 35% [9]. RV‐FWLS values ≥ 20% were considered abnormal [9], and normal values started at −21.5% [10, 11, 12, 13]. While there is no consensus statement regarding a normal range for RV‐4CLS, values starting at −20.1% have been considered normal [11, 12, 13]. Additionally, an sPAP ≥ 36 mmHg and an IVC diameter ≤ 21 mm with an inspiratory collapse > 50% were considered normal [14]. With regard to RV dimensions, a basal RVEDD ranging up to 41 mm [9] and an RA area < 18 cm^2^ [14] were considered normal.

### 2.3 Assessment of Quality of Life

QoL was assessed by using the MLHFQ, which is well established in patients with heart failure [15, 16]. The MLHFQ consisted of 21 questions that could be answered by selecting 5 different options (from 0 to 5). The highest possible score of 105 points represented the worst QoL. The MLHFQ has also been established in German‐speaking patients with a proven reliability and validity [17]. A German translation of the questionnaire can be found in the Supplemental Appendix (Table S1).

### 2.4 Statistical Analysis

Data are presented as continuous (mean ± SD or median with IQR) or categorical (absolute numbers and percentages). Comparison of echocardiographic values within and between the AIC and non‐AIC groups was made by two‐way ANOVA with Tukey's multiple comparison test. Simple logistic analysis was used to determine a possible association between TAPSE/FAC and the final diagnosis of AIC/non‐AIC and simple linear regression was employed to assess the influence of TAPSE/AIC on QoL. *P*‐values were considered statistically significant if *p* < 0.05. Data were analyzed using GraphPad Prism version 10 (San Diego, USA).

## 3 Results

### 3.1 Patient Characteristics

Fifty patients had sinus rhythm until the end of the follow‐up and were analyzed. Forty‐one patients fulfilled the AIC criteria (AIC group), and 9 did not (non‐AIC group). In the AIC group, the mean age was 68.6 ± 11.1 years, 63% of the patients were male, and the mean CHA_2_DS_2_‐VASc score was 3.2 ± 1.7, whereas in the non‐AIC group, the mean age was 65.7 ± 10.9 years, 78% of patients were male, and the mean CHA_2_DS_2_‐VASc score was 3.2 ± 2.1. Baseline medication, CHA_2_DS_2_‐VASc score, New York Heart Association (NYHA) class, and pre‐existing conditions were comparable between the groups. At the time of enrollment, AIC and non‐AIC patients had similar heart rate and LVEF. These and additional baseline characteristics are provided in Table S2.

### 3.2 Right Ventricular Function and Geometric Data

At presentation, TAPSE was reduced in the AIC group (15.4 ± 3.8 mm) but not in the non‐AIC group (19.0 ± 3.1 mm; *p* = 0.0246 vs. AIC). During the follow‐up period, TAPSE increased in the AIC group after successful rhythm restoration (22.8 ± 4.8 after 6 months, *p* < 0.0001 vs. baseline), whereas there was no significant change in the non‐AIC group (21.5 ± 3.9 after 6 months, *p* = 0.786 vs. baseline). Similarly, patients with AIC initially had slightly impaired FAC (34.1% ± 5.5%), whereas FAC was preserved in patients with non‐AIC (37.9% ± 4.4%, *p* = 0.0313 vs. AIC). A significant recovery of FAC in the AIC group was observed after rhythm control therapy (40.9% ± 3.7% after 6 months, *p* < 0.0001 vs. baseline), whereas in the non‐AIC group, FAC did not markedly improve (40.6% ± 3.8% after 6 months, *p* = 0.260 vs. baseline).

**Figure 1:**
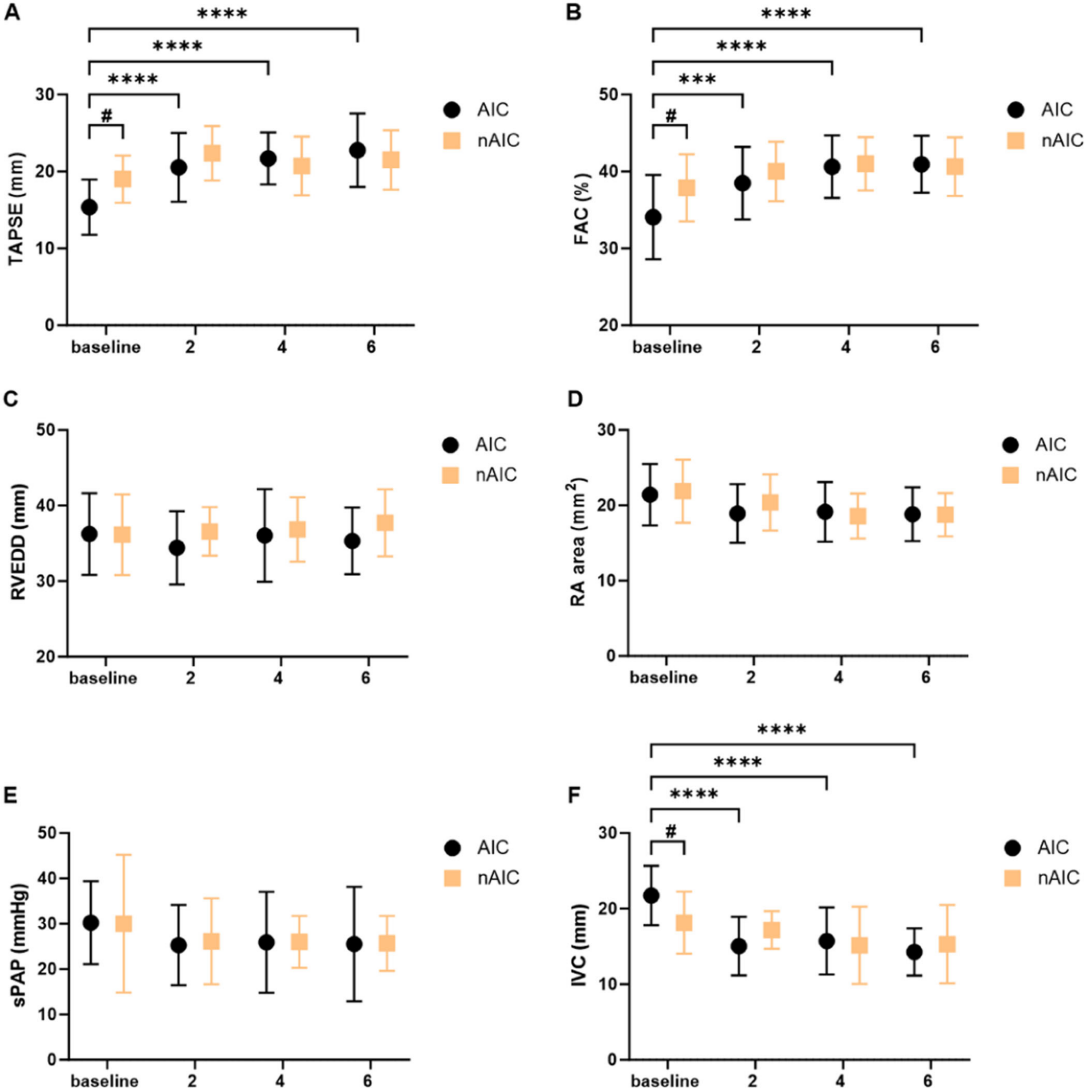
Summary data of RV geometry and function. Patients per group: (A) AIC *n* = 38, nAIC *n* = 8, (B) AIC *n* = 32, nAIC *n* = 8, (C) AIC *n* = 34, nAIC *n* = 7, (D) AIC *n* = 26, nAIC *n* = 8, (E) AIC = 24, nAIC = 6, (F) AIC = 34, nAIC = 7. Data are presented as mean ± SD. AIC, arrhythmia‐induced cardiomyopathy; FAC, fractional area change; IVC, inferior vena cava; nAIC, non‐AIC; RA, right atrial; RVEDD, right ventricular end‐diastolic diameter; sPAP, systolic pulmonary artery pressure, TAPSE, tricuspid annular plane systolic excursion. #*p* < 0.05, ***p < 0.001, ****p < 0.0001.

To assess RV dimensions and to further analyze RV function, basal RVEDD, RA area, sPAP, and IVC were measured and compared between the groups. Basal RVEDD at baseline was normal in patients with AIC and non‐AIC, whereas RA area was slightly enlarged in both groups. The two parameters showed no relevant change over time and no intergroup differences. sPAP was also not elevated in the two groups at the time of study inclusion, whereas IVC was initially dilated in the AIC group (21.7 ± 3.9 mm) but not in the non‐AIC group (18.1 ± 4.1 mm, *p* = 0.03 vs. AIC). IVC in patients with AIC normalized at the 6‐month follow‐up (14.3 ± 3.1 mm, *p* < 0.0001 vs. baseline) (Figure 1).

**Figure 2:**
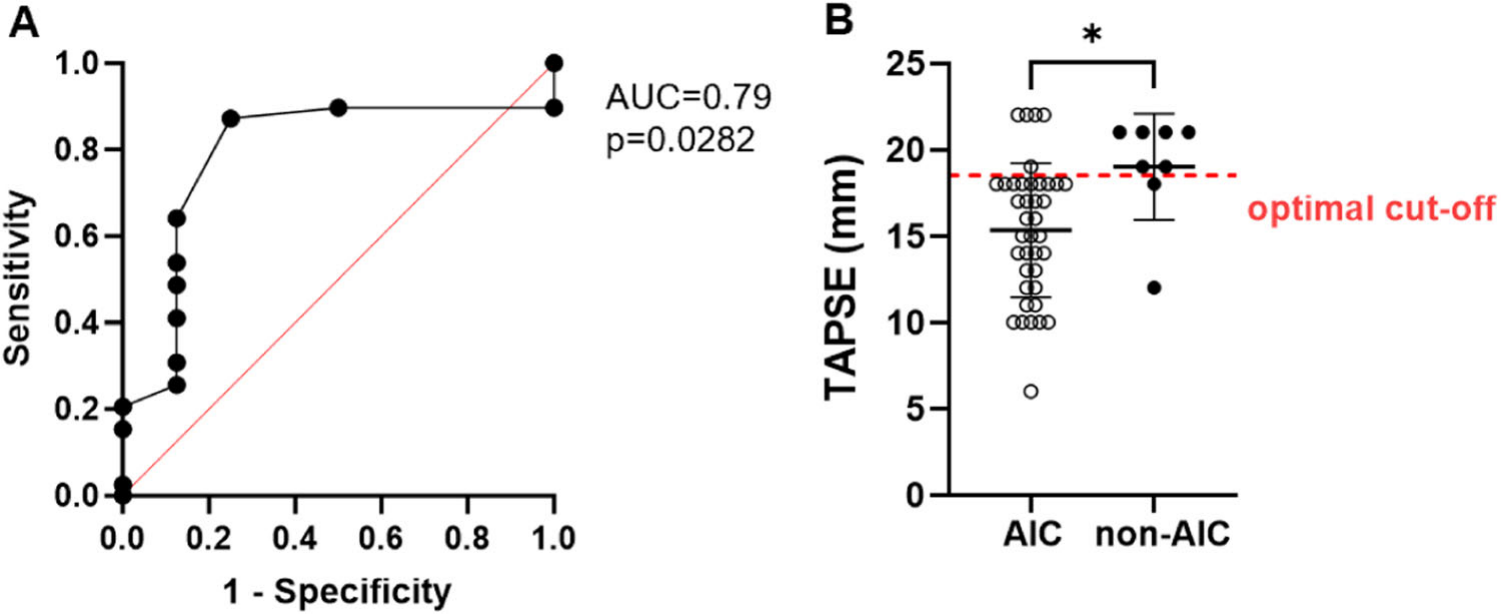
(A) Receiver operating characteristics analysis showing the accuracy of the variable TAPSE in predicting the outcome AIC. (B) Individual data and the optimal cut‐off value (dashed line) of 18.5 mm. AIC, arrhythmia‐induced cardiomyopathy; AUC, area under the curve; TAPSE, tricuspid annular plane systolic excursion. *p < 0.05.

As lower TAPSE and FAC were detected in the AIC group at the time of enrollment, simple logistic analysis was applied to determine whether there was an association between RV function and the final diagnosis of AIC or non‐AIC. FAC showed no predictive value, but TAPSE was identified as a parameter that correlated with the recovery of LVEF (*p* = 0.0091, AUC = 0.79, 95% CI: 0.62 to 0.97) The optimal cut‐off value was a TAPSE of 18.5 mm, with lower values being associated with AIC (Figure 2).

### 3.3 Right Ventricular Strain Measurements

**Figure 3:**
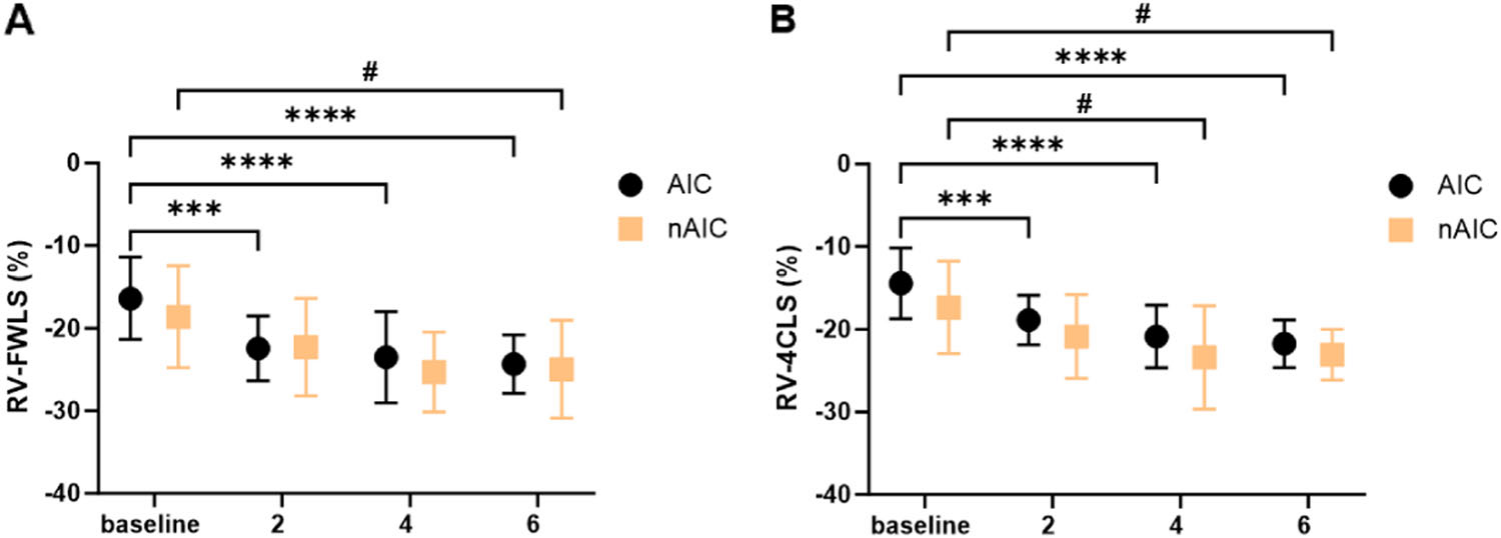
Overview of RV strain measurements. Patients per group: (A) AIC *n* = 26, nAIC *n* = 8, (B) AIC *n* = 26, nAIC *n* = 8. Data are presented as mean ± SD. AIC, arrhythmia‐induced cardiomyopathy; nAIC, non‐AIC; RV‐4CLS, RV four‐chamber longitudinal strain; RV‐FWLS, RV free wall longitudinal strain. #*p* < 0.05, ***p < 0.001, ****p < 0.0001.

As a more sensitive parameter of RV systolic function, RV strain was also analyzed. In contrast to conventional echocardiography, both RV‐FWLS and RV‐4CLS were initially reduced in patients with AIC (RV‐FWLS: −16.4% ± 5.0%; RV‐4CLS: −14.4% ± 4.3%) and non‐AIC (RV‐FWLS: −18.6% ± 6.1%; RV‐4CLS: −17.4% ± 5.6%). RV strain was numerically more impaired in the AIC group, but this difference was not statistically significant. Over time, RV‐FWLS and RV‐4CLS showed a significant improvement in both the AIC (RV‐FWLS: −24.3% ± 3.6% after 6 months, *p* < 0.0001; RV‐4CLS: −21.7% ± 2.9%, *p* < 0.0001) and non‐AIC groups (RV‐FWLS: −25.0% ± 5.9, *p* = 0.0468; RV‐4CLS: −23.0% ± 3.1%, *p* = 0.0208. There were no significant differences between the two groups (Figure 3).

### 3.4 Quality of Life and Association With Right Ventricular Function

**Figure 4:**
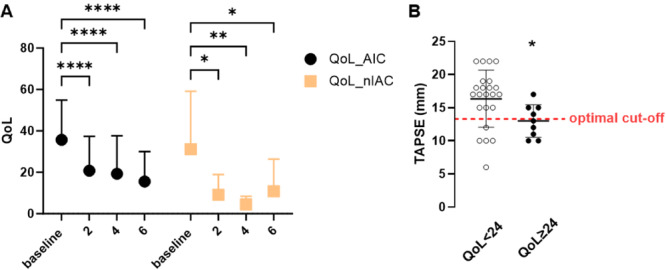
(A) Improvement in QoL was observed in both groups. (B) Individual data with an optimal cut‐off value (dashed line) of 13.3 mm. Patients per group: (A) AIC *n* = 33, nAIC *n* = 7. Data are presented as mean ± SD. AIC, arrhythmia‐induced cardiomyopathy; FAC, fractional area change; nAIC, non‐AIC; QoL, quality of life; TAPSE, tricuspid annular plane systolic excursion. *p < 0.05, **p < 0.01, ****p < 0.0001.

Our assessment of QoL, as measured by the MLHFQ, revealed that patients in both groups were negatively affected by the combination of arrhythmia and heart failure at the time of enrollment. Higher values thereby indicate worse QoL. After rhythm control therapy, QoL improved in patients with AIC (35.7 ± 19.2 vs. 15.6 ± 14.5, *p* < 0.0001) and non‐AIC (31.1 ± 28.1 vs. 10.9 ± 15.2, *p* = 0.0246) after 6 months. There were no significant QoL differences between the groups at the respective time points. In the AIC group, there was also an association between an initially reduced TASPE and an impaired QoL after 6 months (95% CI: −2.949 to −0.1390, *r* = −0,3679, *p* = 0.0323, r^2^ = 0.1354). Further, a QoL value of ≥ 24 after 6 months, which represents moderate to poor QoL [18], was associated with low TAPSE at baseline. The optimal TAPSE cut‐off value was 13.3 mm, with lower values indicating worse QoL (Figure 4).

## 4 Discussion

In this subanalysis of our recently published study of AIC patients, we analyzed for the first time RV function and QoL in patients with AIC. We found that impairment of RV function was more pronounced in patients with AIC than in those without and that TAPSE can be used to predict recovery of LV function. Furthermore, impaired QoL was observed in both groups. Interestingly, lower values for TAPSE at baseline were associated with worse QoL at the end of the study period.

In patients with DCM, it is not uncommon to find a reduced RV function in addition to LVSD. In fact, RV dysfunction is present in approximately one‐third of patients suffering from DCM [5]. In our study, which only included patients with newly diagnosed LVSD, patients with non‐AIC representing a DCM collective had normal values of conventional echocardiographic parameters representative of RV systolic function such as TASPE and FAC. In contrast, TAPSE and FAC were reduced in the AIC group and recovered over time. RV strain analysis, which reveals a more subtle form of RV heart failure [19], showed impairment of RV function in both groups. These findings underscore the importance of recognizing AIC as a specific disease entity that is in part distinct from the “common” form of DCM. In addition, the more pronounced RV dysfunction in AIC suggests a problematic hemodynamic adaption to rapidly emerging stressors such as tachyarrhythmias. The fact that rhythm disturbances such as AFib can lead to RV dysfunction was previously demonstrated in a trial by Yan et al. [20]. In their study, patients with recurrent AFib after electrical cardioversion had markedly impaired TAPSE and FAC [20]. Furthermore, a study in nonischemic patients suffering from LVSD and AFib showed that in patients with AFib, FAC and RV strain were significantly reduced compared with a control group in sinus rhythm [21].

A problem in the diagnostic algorithm of AIC is that the diagnosis can only be made retrospectively after LVEF recovers as a result of rhythm restoration, that is, *diagnosis ex juvantibus* [22, 23]. Currently, there is no generally accepted tool for early diagnosis of AIC, although the search for an early indicator of AIC is of great importance [24], as this would require aggressive rhythm control therapy. Recently, our research group demonstrated that a smaller LVEDD < 56.5 mm at baseline may be predictive of AIC, a finding that should be evaluated in a larger cohort of patients [3]. Besides, the Antwerp score, which includes the parameters known etiology, QRS duration, severe atrial dilatation, and paroxysmal AFib can reliably predict the recovery of LV function after catheter ablation in patients with heart failure [25]. However, it must be noted that the recovery criteria used to validate the Antwerp score were less strict that in our trial, which may lead to an overdiagnosis of patients with an AIC component. The present study identified a correlation between an initial low TAPSE and a final diagnosis of AIC and thus contributes to the current knowledge of possible parameters for early diagnosis of AIC.

Our data show that comparable QoL impairment was present in patients with AIC and non‐AIC and that an improvement in QoL was observed in both groups over time. While it is well established that QoL is impaired in patients with DCM [26], we were able to show for the first time that significant QoL impairment also occurs in a selective AIC cohort in which heart failure is solely due to arrhythmia. This suggests that maintaining sinus rhythm and avoiding AFib, which is accompanied by hemodynamic alterations such as loss of “atrial kick” and reduced cardiac output [27], may be more important for QoL than changes in LVEF. A more pronounced reduction of TAPSE and FAC was associated with worse QoL at the time of the 6‐month follow‐up in all patients in our study; in the AIC group, TASPE also showed a correlation with worse QoL. Thus, TAPSE may be a parameter that could not only potentially predict AIC but also identify patients who are particularly affected by the disease. This is important because reduced TAPSE also has prognostic significance: in a study of patients with congestive heart failure and LVEF < 35% as well as TAPSE ≤ 14 mm, more deaths or emergency cardiac transplantation occurred after a median follow‐up of 16 months [28]. Therefore, patients with more limited TAPSE may require shorter follow‐up periods and patient‐specific strategies to improve QoL.

### 4.1 Limitations

The non‐AIC control group was relatively small due to the unexpectedly high prevalence of AIC in this selective cohort of patients and the intention to investigate the “pure” form of AIC in which arrythmia is the only cause of impaired LV function. Thus, the results might have been different with a higher number of patients in the non‐AIC group. Further, in some cases, RV echocardiographic visibility of the right heart was limited in 4 patients, and thus echocardiographic parameters were not obtained from these patients, which could potentially constitute a bias.

## 5 Conclusion

In patients with atrial tachyarrhythmia with no cause for LVSD other than the arrhythmia itself, initial RV function as measured by conventional echocardiographic parameters and by strain analysis was predominantly reduced in the AIC group. QoL was reduced similarly in AIC and non‐AIC patients. TAPSE at baseline correlated not only with the diagnosis of AIC but also with QoL at 6 months. These findings should be evaluated in a larger cohort of patients, particularly TAPSE as a parameter that could early recognize AIC and identify patients with poor QoL.

## Disclosure

The authors have nothing to report.

## Conflicts of Interest

The authors declare no conflicts of interest.

## Supporting information

Supporting information.

## Data Availability

The primary data analyzed in this study are not publicly available; some parameters that do not contain personal information can be provided by the corresponding author upon reasonable request.
