# Supplementary material for: Right Ventricular Systolic Dysfunction Predicts Recovery of Left Ventricular Systolic Function and Reduced Quality of Life in Patients With Arrhythmia‐Induced Cardiomyopathy
Source: Clin Cardiol. 2025 Feb 22;48(2):e70070. doi: 10.1002/clc.70070 (PMC11845871; doi:10.1002/clc.70070)
Supplement: Supplementary file 1 — Supporting information. [file CLC-48-e70070-s001.docx]

**Supplemental Appendix**

**Supplementary Figure 1:**


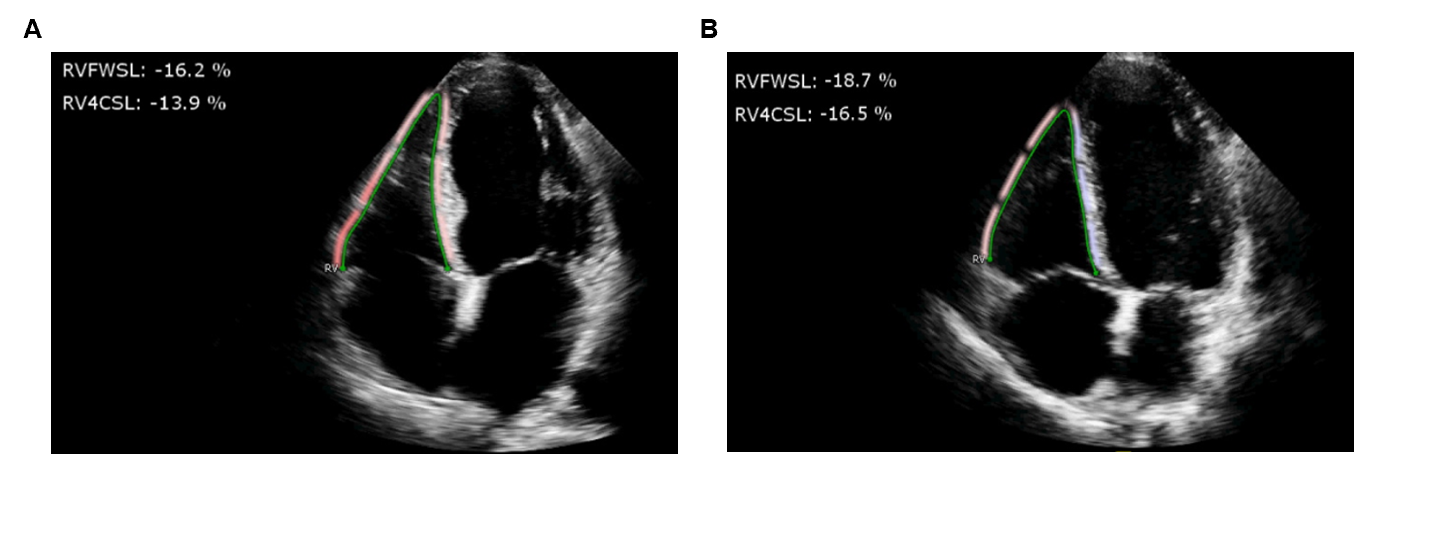


Supplementary Figure 1: Representative strain measurements of patients with (A) AIC and (B) non-AIC at the time of enrollment. RV-FWLS=RV free wall longitudinal strain, RV-4CLS = RV four-chamber longitudinal strain.

**Supplementary Table 1:**

*Minnesota Quality of Life Questionnaire – German Version*

Fragebogen „Lebensqualität bei Herzschwäche“

Die folgenden 21 Fragen beschäftigen sich mit der Beeinträchtigung Ihres Lebens bezüglich Ihrer Herzschwäche im vergangen Monat (4 Wochen). Bitte kreisen Sie nach **jeder Frage** 0, 1, 2, 3, 4 oder 5 ein, um zu zeigen – wie sehr Ihr Leben beeinträchtigt ist. Wenn eine Frage nicht zutrifft, kreisen Sie 0 ein.

| **Hat Ihre Herzschwäche/Herzleiden Sie im vergangenen Monat (4 Wochen) in den folgenden Punkten von Ihrer gewohnten**  **Lebensweise abgehalten -­** | | | | **trifft nicht zu** 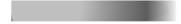 | 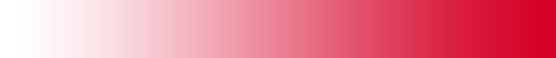 | | | | **trifft definitiv**  **zu** |
| --- | --- | --- | --- | --- | --- | --- | --- | --- | --- |
| 1. aufgrund einer Schwellung der Knöchel oder Beine? | | | | 0 | 1 | 2 | 3 | 4 | 5 |
| 2. Mussten Sie sich tagsüber vermehrt hinsetzen oder hinlegen? | | | | 0 | 1 | 2 | 3 | 4 | 5 |
| 3. Hatten Sie Probleme beim Gehen oder Treppensteigen? | | | | 0 | 1 | 2 | 3 | 4 | 5 |
| 4. Haben sie eine Beeinträchtigung bei der Haus – oder Gartenarbeit bemerkt? | | | | 0 | 1 | 2 | 3 | 4 | 5 |
| 5. Gab es Schwierigkeiten das Haus zu verlassen? | | | | 0 | 1 | 2 | 3 | 4 | 5 |
| 6. Hatten Sie Schwierigkeiten nachts gut zu schlafen? | | | | 0 | 1 | 2 | 3 | 4 | 5 |
| 7. Fielen Ihnen Unternehmungen mit Ihrer Familie oder Freunden schwer? | | | | 0 | 1 | 2 | 3 | 4 | 5 |
| 8. Hatten sie aufgrund dessen Schwierigkeiten Ihren Lebensunterhalt zu verdienen? | | | | 0 | 1 | 2 | 3 | 4 | 5 |
| 9. Gestaltete sich Ihre Freizeit schwierig nachzugehen, Sport zu betreiben, . . .)? | (z. | B.: | Hobbies | 0 | 1 | 2 | 3 | 4 | 5 |
| 10. Machte es Ihre Sexualleben mit Ihrem Partner/in schwierig? | | | | 0 | 1 | 2 | 3 | 4 | 5 |
| 11. Hatten sie weniger Lust auf Ihr Lieblingsessen oder im Allgemeinen weniger gegessen? | | | | 0 | 1 | 2 | 3 | 4 | 5 |
| 12. aufgrund einer Kurzatmigkeit? | | | | 0 | 1 | 2 | 3 | 4 | 5 |
| 13. aufgrund von Müdigkeit, Abgeschlagenheit oder Kraftlosigkeit? | | | | 0 | 1 | 2 | 3 | 4 | 5 |
| 14. aufgrund eines Krankenhausaufenthaltes? | | | | 0 | 1 | 2 | 3 | 4 | 5 |
| 15. aufgrund der Kosten, welche Sie für Medikamente ausgegeben haben? | | | | 0 | 1 | 2 | 3 | 4 | 5 |
| 16. aufgrund von Nebenwirkungen einer Behandlung (z. B.: Medikamente, . .)? | | | | 0 | 1 | 2 | 3 | 4 | 5 |
| 17. aufgrund des Gefühls Ihrer Familie oder Freunden zur Last zu fallen? | | | | 0 | 1 | 2 | 3 | 4 | 5 |
| 18. Haben Sie einen Verlust der Selbst-­Kontrolle bemerkt? | | | | 0 | 1 | 2 | 3 | 4 | 5 |
| 19. aufgrund von Zweifel oder Angst? | | | | 0 | 1 | 2 | 3 | 4 | 5 |
| 20. Hatten Sie diesbezüglich Konzentrationsschwieirgkeiten oder Probleme sich an Dinge zu erinnern? | | | | 0 | 1 | 2 | 3 | 4 | 5 |
| 21. Weil Sie sich niedergeschlagen/deprimiert gefühlt hatten? | | | | 0 | 1 | 2 | 3 | 4 | 5 |

**Supplementary Table 2: Baseline characteristics**

|  | AIC group (n=41) | Non-AIC group (n=9) |
| --- | --- | --- |
| Age | 68.6 ± 11.1 | 65.7 ± 10.9 |
| Sex, male | 26 (63) | 7 (78) |
| Weight (kg) | 89.0 ± 21.0 | 88.8 ± 34.0 |
| Height (cm) | 172.6 ± 10.8 | 173.7 ± 10.3 |
| BMI (kg/m²) | 29.5 ± 5.3 | 28.8 ± 8.2 |
| BSA (m²) | 2.1 ± 0.3 | 2.1 ± 0.4 |
| Diabetes | 10 (24) | 0 (0) |
| Arterial hypertension | 32 (78) | 6 (67) |
| Renal insufficiency | 12 (29) | 3 (33) |
| CHA_2_DS_2_-VASc score | 3.2 ± 1.7 | 3.2 ± 2.1 |
| Smoker status | 6 (15) | 1 (11) |
| Previous PCI/CABG | 6 (14) | 0 (0) |
| NYHA class | 2.9 ± 0.7 | 3.0 ± 0.7 |
| QoL (points) | 36.6 ± 19.8 | 27.8 ± 25.9 |
| Biomarkers |  |  |
| hs-cTnT (ng/L) | 27.0 ± 22.2 | 22.9 ± 16.8 |
| NT-proBNP (pg/mL) | 2689 (1089-6038) | 2381 (1527-10462) |
| ECG and Echocardiography |  |  |
| Heart rate (beats/min) | 127.1 ± 16.3 | 120.7 ± 11.2 |
| Atrial fibrillation | 28 (68) | 9 (100) |
| Atrial flutter | 13 (32) | 0 (0) |
| LVEF (%) | 35.4 ± 8.2 | 37.0 ± 9.5 |
| LVEDD (mm) | 53.4 ± 6.2 | 60.4 ± 4.1 |
| LVESD (mm) | 43.4 ± 6.9 | 49.8 ± 6.0 |
| Mitral regurgitation (grade) | 1.5 ± 0.7 | 2.1 ± 1.1 |
| LA area (cm²) | 26.5 ± 5.1 | 29.9 ± 7.3 |
| LAVI (mL/m²) | 48.3 ± 16.7 | 62.5 ± 26.4 |
| TAPSE (mm) | 15.4 ± 3.8 | 19.0 ± 3.1 |
| FAC (%) | 34.1 ± 5.5 | 37.9 ± 4.4 |
| RV-FWLS (%) | -16.4 ± 5.0 | -18.6 ± 6.1 |
| RV-4CLS (%) | -14.4 ± 4.3 | -17.4 ± 5.6 |
| RVEDD (mm) | 36.2 ± 5.4 | 36.1 ± 5.3 |
| RA area (cm^2^) | 21.4 ± 4.1 | 21.9 ± 4.2 |
| sPAP (mmHg) | 30.2 ± 9.2 | 30.0 ± 15.2 |
| IVC (mm) | 21.7 ± 3.9 | 18.1 ± 4.1 |
| Medication |  |  |
| Beta-blocker | 34 (83) | 7 (78) |
| ACEI/ARB | 30 (73) | 5 (56) |
| ARNI | 6 (15) | 1 (11) |
| Mineralocorticoid antagonist | 7 (17) | 1 (11) |
| Calcium antagonist | 8 (20) | 3 (33) |
| Diuretics | 25 (61) | 5 (56) |
| Digoxin | 3 (7) | 0 (0) |
| Amiodarone | 2 (5) | 0 (0) |
| Statin | 10 (24) | 4 (44) |
| ASA | 3 (7) | 0 (0) |
| P2Y_12_ inhibitor | 2 (5) | 0 (0) |
| NOAC | 27 (66) | 5 (56) |
| VKA | 2 (5) | 1 (11) |

Values are presented as mean ± SD or n (%), except for NT-proBNP with median (IQR). ACEI = angiotensin-converting-enzyme inhibitors; AIC = arrhythmia-induced cardiomyopathy; ARB = angiotensin receptor blocker; ARNI = angiotensin receptor/neprilysin inhibitor; ASA = acetylsalicylic acid; BMI = body mass index; BSA = body surface area; CABG = coronary artery bypass graft; hs-cTnT = high-sensitivity cardiac troponin T; ECG = electrocardiography; FAC = fractional area change; IVC = inferior vena cava; NYHA = New York Heart Association; NT-proBNP = N-terminal prohormone of brain natriuretic peptide; LVEF = left ventricular ejection fraction; LVEDD = left ventricular end-diastolic diameter; LVESD = left ventricular end-systolic diameter; LA = left atrial; LAVI = left atrial volume index; NOAC = new oral anticoagulants; PCI = percutaneous coronary intervention; QoL = quality of life; RA = right atrial; RV-4CSL = RV four-chamber longitudinal strain; RV-FWSL = RV free wall longitudinal strain; RVEDD = right ventricular end-diastolic diameter; sPAP = systolic pulmonary artery pressure; TAPSE = tricuspid annular plane systolic excursion; VKA = vitamin K antagonist.
